# “What Women Like”: Influence of Motion and Form on Esthetic Body Perception

**DOI:** 10.3389/fpsyg.2012.00235

**Published:** 2012-07-09

**Authors:** Valentina Cazzato, Serena Siega, Cosimo Urgesi

**Affiliations:** ^1^Dipartimento di Scienze Umane, Università di UdineUdine, Italy; ^2^Istituto di Ricovero e Cura a Carattere Scientifico “E. Medea,” San Vito al TagliamentoPordenone, Italy

**Keywords:** esthetic judgments, body perception, implied motion, body shape, body posture

## Abstract

Several studies have shown the distinct contribution of motion and form to the esthetic evaluation of female bodies. Here, we investigated how variations of implied motion and body size interact in the esthetic evaluation of female and male bodies in a sample of young healthy women. Participants provided attractiveness, beauty, and liking ratings for the shape and posture of virtual renderings of human bodies with variable body size and implied motion. The esthetic judgments for both shape and posture of human models were influenced by body size and implied motion, with a preference for thinner and more dynamic stimuli. Implied motion, however, attenuated the impact of extreme body size on the esthetic evaluation of body postures, while body size variations did not affect the preference for more dynamic stimuli. Results show that body form and action cues interact in esthetic perception, but the final esthetic appreciation of human bodies is predicted by a mixture of perceptual and affective evaluative components.

## Introduction

Esthetic experience seems to be a universal phenomenon common to humans throughout the ages. From a neurocognitive point of view, it can be described as the ability to assign a particular reward, hedonic value to a given stimulus or to one of its features, thus involving an evaluative judgment. In particular, the esthetic evaluation of stimuli may be defined by an objective, shared attribute regarding the perceptual properties of the stimulus (beauty) and a more subjective, individual attribute concerning the personal attitude of the observer toward the stimulus or its features (liking; Calvo-Merino et al., 2008; Cela-Conde et al., 2011). In terms of the different stimuli we perceive, the esthetic evaluation of the human body has a particular importance for our survival, being strictly connected with reproductive behavior, in so far as attractiveness is an essential attribute of body esthetic experience and, indeed, the body plays a crucial role in attracting and selecting a partner. Considerable evidence has been accumulated in recent years supporting the notion that both facial and bodily physical attractiveness are “health certifications” and thus represent honest signals of phenotypic and genetic quality (Grammer et al., 2003).

A special place as beauty “signaling” is assumed by body movements. Movement does not only convey information on gender identification, however, which is one of the major sources for attractiveness ratings (Johnson and Tassinary, 2007). Simply by observing others’ body movements, we are able to identify their effort, intention, and deception (Grezes et al., 2004). Furthermore, studies have shown that symmetry and consistency of movements differ between healthy and sick individuals (Escós et al., 1995) and may also be related to the sex hormone profile (Hampson and Kimura, 1988; Grammer et al., 1997).

Apart from motion, body form exerts a strong influence on esthetic body judgments. Studies have suggested two potentially important perceptual cues for female bodies’ physical attractiveness: body fat distribution [the waist-to-hip ratio (WHR)] and overall body fat [often estimated by the body mass index (BMI; Singh, 1993; Tovée et al., 1998; Fan et al., 2004; Smith et al., 2007a; Streeter and McBurney, 2007; Holliday et al., 2011]. Low values of WHR index are related to optimal fat distribution as an expression of high fertility (Zaadstra et al., 1995), and changes in BMI have a strong impact on health (Manson et al., 1995; Willet et al., 1995) and reproductive potential (Reid and Van Vugt, 1987; Frisch, 1988; Lake et al., 1997).

The ideal WHR and BMI, however, seem to be influenced by socio-cultural factors, including media exposure to thin models. In particular, a tendency toward a beauty ideal of extreme thinness has been documented in Western societies (Collins, 1991; Feingold and Mazzella, 1998; Kostanski and Gullone, 1998; Pine, 2001; Truby and Paxton, 2002). Importantly, culturally mediated beauty ideals include not only thinness but also an extremely fit and toned appearance (Homan et al., 2012). Furthermore, exercise, more than dieting, is emphasized as a means of losing weight in magazine articles (Luff and Gray, 2009), thus suggesting a strong link between thinness and fitness ideals. The internalization of such ideals has strong implications for the well-being and body satisfaction of adolescent and adult individuals (Kenardy et al., 2001; McCabe and Ricciardelli, 2001) and may represent a critical risk factor for eating disorders (Thompson and Stice, 2001; Bessenoff, 2006). Crucially, although patients with eating disorders present specific alterations in the visual perception of human body form (Urgesi et al., 2012), these may extend also to the processing of others’ movements (Vocks et al., 2007). Thus, body morphology and movement perception may interact in body esthetic perception.

The visual perception of body form and action involves specific neural structures that are at least partially segregated from those involved in the visual processing of other objects and even of human faces (Peelen and Downing, 2005). Observation of body actions employs a large fronto-temporo-parietal system which includes not only visual areas, such as the superior temporal sulcus (Grossman and Blake, 2002), but also motor areas (Rizzolatti and Craighero, 2004), even when the actions are only implied by static human postures (Urgesi et al., 2006, 2007b; Candidi et al., 2008). On the other hand, the visual processing of body forms relates to a lateral occipito-temporal area, referred to as *extrastriate body area* (EBA; Downing et al., 2001; Urgesi et al., 2004, 2007a; Moro et al., 2008), and a medial fusiform region, known as fusiform body area (FBA; Peelen and Downing, 2005).

Recently, a series of studies provided evidence that not only visual but also motor areas are involved in esthetic body perception (Di Dio et al., 2007; Calvo-Merino et al., 2008, 2010). Di Dio et al. (2007) found greater activation in the occipital, insular, and premotor cortices during observation of images of statues obeying the golden section, a principle of spatial proportion felt to be classically beautiful, than during observation of statues not following this principle. In a similar vein, Cross et al. (2011) reported a greater activation of EBA cortices during observation of dance moves when participants view movements they rate as both esthetically pleasing and difficult to reproduce. These results suggest a strong link between simulative processing and esthetic perception of dance (Freedberg and Gallese, 2007).

Capitalizing on such neural and behavioral evidence, here we sought to investigate the contribution of implied motion and form to esthetic body perception. In line with previous studies (Calvo-Merino et al., 2010), we investigated two aspects of esthetic evaluation: one related to the attribution of an “intrinsic perceptual property” to the stimulus (beauty) and the other related to the “observer’s attitude” to the stimulus (liking). Furthermore, we also investigated how the esthetic evaluation of human bodies is related to the attractiveness of their physical and psychological features (Grammer et al., 2003). In particular, we focused on subjective visual analog scale (VAS) ratings of “beauty,” “liking,” and “attractiveness” about adult female and male bodies whose width was varied to apparently increase or diminish their body size. Furthermore, the implied movement of each body was manipulated by the use of static or dynamic poses in order to obtain information about the influences of motion on the subjective evaluations of participants. We also collected a series of ratings on the perceived physical and psychological features of the models to investigate the contribution of perceptual and affective factors to esthetic body perception. Importantly, whereas previous studies have focused on the attractiveness of female bodies perceived by male and, more rarely, female observers (Wack and Tantleff-Dunn, 2008; Yamamiya and Thompson, 2009) we investigated the esthetic perception of male and female bodies by female observers (Cornelissen et al., 2009; Doyle, 2009; Brooks et al., 2010). We focused on female observers as previous studies have shown greater internalization of thinness ideals, body dissatisfaction, and risk of eating disorders in women than men (Johansson et al., 2005; Brown and Slaughter, 2011). We hypothesized that the esthetic judgments attractive, beauty, and liking would be modulated by the thinness and fitness ideals irrespective of the models’ gender. In addition, we expected an influence of implied motion on perception of body size (i.e., slim vs. fat bodies), as motion seems to be a relevant cue for the esthetic judgments of human bodies (Grezes et al., 2004; Johnson and Tassinary, 2007).

## Materials and Methods

### Participants

A convenience sample of 86 female students from the University of Udine, Italy participated in the experiment in return for course credits. No participants reported any current neurological or psychiatric disorders. Four subjects were excluded because they reported past history of eating disorder disease. Voluntary informed consent was obtained from each participant in accordance with the ethics committee of the Scientific Institute (IRCCS) Eugenio Medea (Italy). All subjects but eight were right-handed and all reported normal or corrected-to-normal vision. Participants’ BMI was estimated from self-report measures of weight and height. Two additional subjects with a BMI >30 kg/m^2^ were discarded from the main analysis. Therefore, for the final analyses we retained a sample of 80 female participants, with a mean age of 24.23 years (SD = 4.44; range: 20–42) and a self-reported mean BMI of 21.23 kg/m^2^ (SD = 2.24; range: 16.46–26.73).

### Stimuli and procedure

#### Stimuli

**Figure 1:**
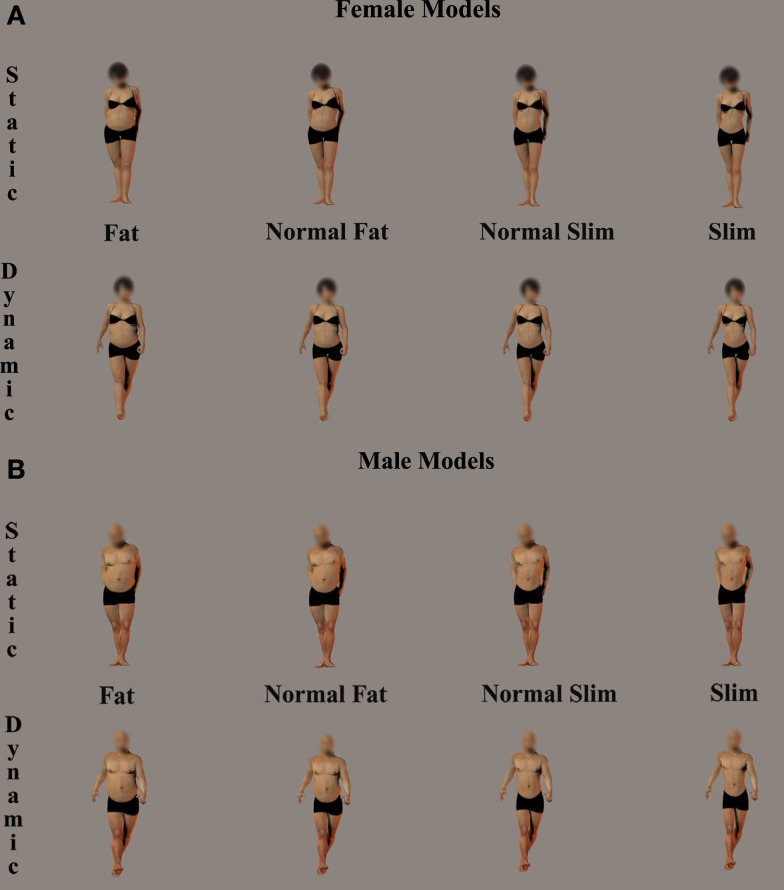
**Examples of female and male virtual models used in the study**. The examples depict, from left to right, the body weight variation of either static or dynamic female **(A)** and male **(B)** models from extreme fat, normal fat, to normal slim, and extreme slim.

To control systematically for the size and implied motion of our body stimuli, we used Poser Pro 2010 (e-frontier, Santa Cruz, CA, USA) and created colored virtual human models selected from a Poser’s default database (Alyson, Maria, Sydney, James, Ryan, and Torno). The Poser rendering software allowed us to create alterable 3-D human figure models with the standard “emaciated” and “heavy” settings supplied by the software. The widths of the bodies were progressively increased or decreased to create a set of four body weights for each model (i.e., extremely fat, normally fat, normally slim, and extremely slim). Crucially, the manipulation of the anthropomorphic body measures was obtained by using the Poser function to enlarge or reduce body parts coherently (i.e., thighs, belly), producing stimuli with naturalistic body proportions. Importantly, each female and male model was rendered in six different daily poses, three static (e.g., standing) and three moving postures (e.g., running, walking). The models were pictured standing in frontal view against a gray background and wearing identical black underwear. Photorealistic textures were applied and the images were enhanced with global illumination (see Figure 1). Finally, in order to avoid the influence of facial features, the pictures were imported into Adobe Photoshop 7.0 (Adobe System Inc., CA, USA; http://www.adobe.com) and a circular region around the face was scrambled. A total of 144 stimuli were created: six models (three males and three females) × six postures (three static, three dynamic) × four body weights (extremely fat, normally fat, normally slim, and extremely slim).

#### Subjective evaluation scales

The experiment was created with E-Prime software (version 1.1, Psychology Software Tools, Inc., Pittsburgh, PA, USA). The questionnaire consisted of brief instructions followed by requests for the participants’ demographic details (age, handedness, weight, and height) and the rating scale trials. Each trial started with the appearance of a black central fixation cross presented on a white light background. After 500 ms, a question asking for the esthetic judgment appeared on the top of the screen, while an image depicting a male or female model appeared on the left side of the screen and subtended an 18.80° × 23.21° region. A VAS presented on the right side of the screen allowed the participants to express their ratings by positioning the mouse along a 100-mm line. The up- and downward anchor words of the VAS scale were presented for each question.

In two different blocks, we asked participants to rate each picture by focusing on the body shape and on the body posture of each model. The order of presentation of each block was balanced across participants. The “Body Shape block” included nine questions about: beauty (beautiful/ugly; in Italian *bello/brutto*), liking (like/dislike; *mi piace/non mi piace*), attractiveness (attractive/repulsive; *attraente/repulsivo*), emotion (positive/negative; *positivo/negativo*), proportion (proportioned/disproportioned; *proporzionato/sproporzionato*), athleticism (athletic/weak; *atletico/gracile*), fatness (slim/fat; *magro/grasso*), perceived health (health/sick; *in salute/malato*), and similarity to the self-body (similar/dissimilar; *mi somiglia/non mi somiglia*). The “Body Posture Block” included six esthetic questions about: beauty (beautiful/ugly; *bello/brutto*), liking (like/dislike; *mi piace/non mi piace*), emotion (positive/negative; *positivo/negativo*), harmony (harmonic/disharmonic; *armonioso/disequilibrato*), implied motion (dynamic/static; *movimento/statico*), and reproducibility of the movements (easy/difficult to imitate; *facile da imitare/difficile da imitare*). For example, when rating the beauty dimension about the “Body Shape,” the participants were asked: “How beautiful do you think the model is?” and they had to click the mouse along a continuous line from “very beautiful” to “very ugly.” When participants were requested to rate the beauty of the “Body Posture,” they were asked: “How beautiful do you think the posture is?”; in this case the participants had to rate the beauty of the posture alone and ignore the body shape. No time limit was fixed for the response, but participants were asked to express their ratings as quickly as possible.

From a dataset of 144 stimuli, each participant evaluated 36 stimuli. In order to balance the total number of stimuli, we assigned each participant to one of four lists in which we presented all the six models (three male, three female) in all six postures (three dynamic, three static). All four body weight levels (extremely fat, normally fat, normally slim, extremely slim) were presented for each participant, but each participant rated each model for only two body weight levels. Thus, all participants rated all body weight levels but applied them to different models. Each participant provided 324 VAS ratings (9 questions × 36 stimuli) for the Body Shape Block and 216 (6 questions × 36 stimuli) VAS ratings for the Body Posture Block, for a total of 540 ratings per participant.

#### Design and statistical analysis

Statistical analyses were run with Statistica 8.0 software (StatSoft Inc., Tulsa, OK, USA). First, we ascertained whether our manipulation of the models’ body weight was parametrically reflected by the judgments of body heaviness provided by our female sample. Second, we investigated the factors influencing implied motion judgment of postures to evaluate whether these subjective judgments were affected by implied motion and body weight manipulations. Third, we examined attractiveness VAS scores for body shape to find the influence of implied motion and body weight cues on esthetic judgments. Thus, three separate 4 × 2 analyses of variance (ANOVAs) with Body weight (extreme fat, normal fat, normal slim, and extreme slim) and Implied Motion (Static, Dynamic) as within-subjects variables were run on Body Shape fatness (i.e., “How fat do you think the model is?”) and attractiveness (i.e., “How attractive do you think the model is?”) judgments and on the Body Posture implied motion judgments (i.e., “How much do you think the posture evokes motion?”). Then, the VAS judgments of beauty and liking of virtual model stimuli were entered into a series of 2 × 4 × 2 ANOVAs with block (Shape, Posture), body weight (extremely fat, normally fat, normally slim, and extremely slim), and implied motion (Static, Dynamic) as within-subjects variables.

Finally, in order to identify which dimensions were associated with esthetic judgment of attractiveness, beauty, and liking for each stimulus, we conducted standard multiple regression analyses separately for the Body Shape and Body Posture blocks. For each stimulus, we computed the mean judgment scores provided by all participants in response to each question. For the Body Shape block, the VAS judgments of attractiveness, beauty, and liking were separately entered as dependent variables, and the VAS judgments of emotions, symmetry, athleticism, fatness, health, and similarity were entered as independent variables. In a similar vein, for the Body Posture block, the VAS judgments of beauty and liking were separately entered as dependent variables, and the VAS judgments of emotion, harmony, implied motion, and reproducibility were entered as independent variables. The assumptions for multiple regression analysis were met as there were linear relationships between the dependent and the independent variables, and the variables were also checked for homoscedasticity and collinearity. The significance threshold was set at *p* < 0.05 in all statistical tests. The source of all significant ANOVA interactions was analyzed with the Newman–Keuls *post hoc* test. All data are reported as Mean ± Standard Error of the Mean (SEM).

## Results

### Influence of body size and motion on heaviness perception of the models’ body shape

We investigated how the fatness rating of human models in the Body Shape block was influenced by the manipulation of body weight and body action cues. The ANOVA revealed a main effect of body weight [*F*(3, 237) = 275.79; *p* < 0.001; η ${\text{η}}_{p}^{2}$ = 0.777] and implied motion [*F*(1, 79) = 46.09; *p* < 0.001; η ${\text{η}}_{p}^{2}$ = 0.368]. Thus, the participants’ judgments were influenced not only by the manipulation of body weight, but also by the implied motion, with models with the same body weight being perceived as thinner when presented in a dynamic (52.9 ± 1.42) than in a static posture (56.1 ± 1.32). Importantly, the two-way interaction of implied motion and body weight was also significant [*F*(3, 237) = 8.43; *p* < 0.001; η ${\text{η}}_{p}^{2}$ = 0.096; see Table 1). *Post hoc* comparisons showed that extremely and normally fat stimuli were always judged fatter than extremely and normally slim stimuli, independently of implied motion levels (all *p*s < 0.001). Furthermore, extremely fat models were rated fatter than normally fat models, whereas extremely slim models were rated thinner than normally slim models (all *p*s < 0.001). This confirmed the successful experimental manipulation of body weight. Although, however, extremely and normally fat and normally slim models were judged thinner when presented in a dynamic than in a static posture (all *p*s < 0.001), no difference between dynamic and static postures was observed for extremely slim models (*p* = 0.577).

**Table 1:** **Mean and standard error of mean (in brackets) for fatness judgment of body shape as a function of implied motion (static/dynamic) and body weight (extreme fat, normal fat, normal slim, and extreme slim)**.

Body weight	Static	Dynamic
Extreme fat	81.80 (1.51)	76.79 (1.69)
Normal fat	65.93 (1.36)	62.64 (1.51)
Normal slim	47.94 (2.19)	43.88 (2.17)
Extreme slim	28.68 (1.93)	28.29 (1.96)

### Influence of body size and movement on implied motion perception of the models’ postures

We investigated whether the perception of implied motion in the models’ postures was influenced by the manipulation of weight and motion bodily cues. The ANOVA on the Body Posture implied motion judgments showed a significant main effect of implied motion [*F*(1, 79) = 769.21; *p* < 0.001; η ${\text{η}}_{p}^{2}$ = 0.907] with dynamic postures (80.22 ± 1.03) implying more movement than static postures (24.58 ± 1.86). Furthermore, the main effect of body weight was significant [*F*(3, 237) = 3.2; *p* = 0.024; η ${\text{η}}_{p}^{2}$ = 0.039], showing that extremely slim models (53.44 ± 1.23) were judged as implying more motion than normally fat models (51.47 ± 1.24; *p* = 0.047), whereas no differences were found for the other body weights (all *p*s > 0.058). The manipulation of body weight and implied motion exerted independent effects on implied motion perception, as the interaction failed to reach the significance level [*F*(3, 237) = 0.999; *p* = 0.394; η ${\text{η}}_{p}^{2}$ = 0.012].

Taken together, these results show that, whereas the amount of implied motion of the postures strongly affected the perception of body fatness, with the notable exception of extremely slim models, body fatness did not affect the perception of implied motion evoked by model postures.

### Influence of body size and motion on attractiveness judgments of the models’ body shape

**Figure 2:**
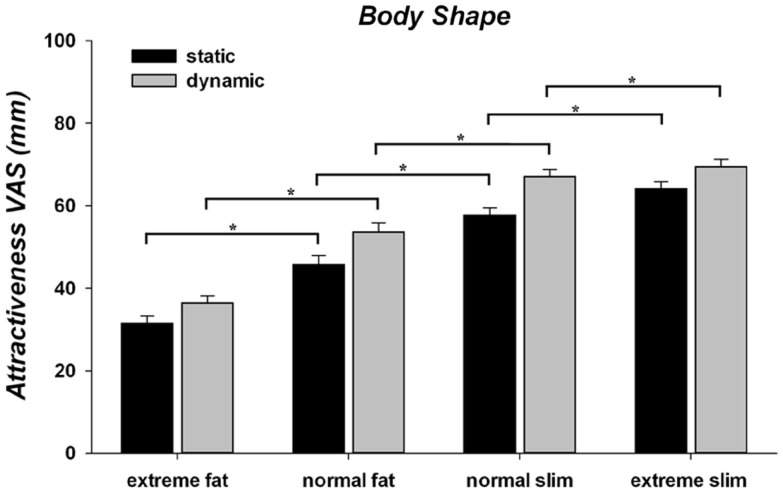
**Mean and standard error of mean for attractiveness VAS ratings of human models body shape as a function of implied motion (static/dynamic) along the four categories of body weight (extreme fat/normal fat/normal slim/extreme slim)**. *p < 0.05.

The ANOVA for the attractiveness judgments (Figure 2) revealed that the main effect of body weight [*F*(3, 237) = 105.88; *p* < 0.001; η ${\text{η}}_{p}^{2}$ = 0.573] and implied motion [*F*(1, 79) = 47.56; *p* < 0.001; η ${\text{η}}_{p}^{2}$ = 0.376] were both significant. The two-way interaction between implied motion and body weight was also significant [*F*(3, 237) = 4.85; *p* = 0.003; η ${\text{η}}_{p}^{2}$ = 0.058]. *Post hoc* pair-wise comparisons showed that along the four body weights, dynamic models were always judged more attractive than their static versions (all *p*s < 0.001). Furthermore, both static and dynamic stimuli were judged more attractive when rendered with a thinner than with a fatter body weight (all *p*s < 0.05). However, for the extreme fat to normal slim weight static bodies were judged more attractive than their fatter dynamic versions, while dynamic normal slim stimuli were more attractive than static extreme slim stimuli. This suggests that implied motion had a greater effect than body weight on the attractiveness judgments of models with a normal or extreme slim body.

### Influence of body size and motion on beauty judgments of body shape and posture

**Figure 3:**
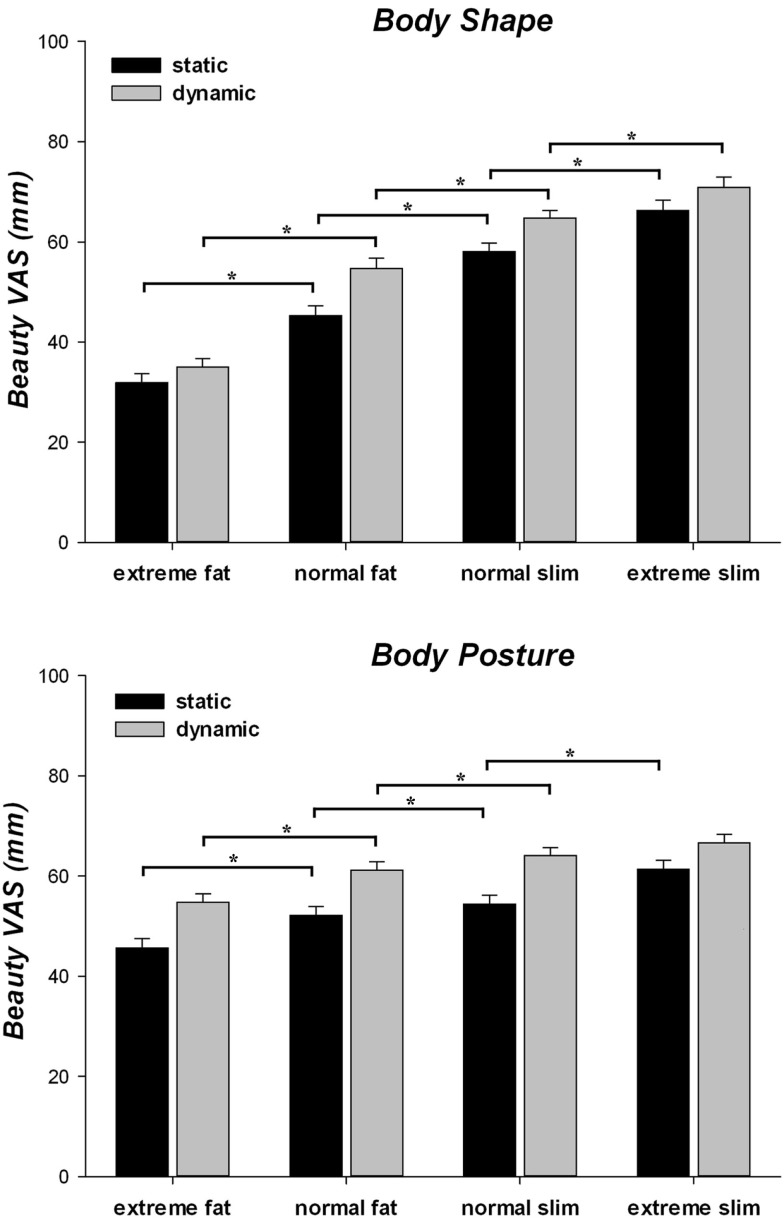
**Mean and standard error of mean for beauty subjective rating of human models as a function of body shape and posture block, implied motion (static/dynamic) along the four categories of body weight (extreme fat/normal fat/normal slim/extreme slim)**. *p < 0.05.

The ANOVA on the esthetic evaluation of beauty (Figure 3) revealed that all main effects were significant [all *F*s > 17.25; *p*s < 0.001; η ${\text{η}}_{p}^{2}$ > 0.0179], with overall higher beauty judgments to slim and dynamic stimuli than to fat and static stimuli. Furthermore, the beauty judgments were higher when participants evaluated the posture than the model depicted in the stimuli. The two-way interactions between block and body weight [*F*(3, 237) = 45.72; *p* < 0.001; η ${\text{η}}_{p}^{2}$ = 0.367], and between body weight and implied motion [*F*(3, 237) = 5.55, *p* = 0.001; η ${\text{η}}_{p}^{2}$ = 0.066] were also significant, further qualified by a significant three-way interaction [*F*(3, 237) = 3.56, *p* = 0.015; η ${\text{η}}_{p}^{2}$ = 0.043]. *Post hoc* pair-wise comparisons showed that the presentation in a dynamic vs. static posture always increased the beauty judgments for the body shape and body posture of fat and slim models (all *p*s < 0.001). Furthermore, in both body shape and body posture blocks and for both static and dynamic postures, normal fat stimuli were judged as less beautiful than normal and extreme slim stimuli and more beautiful than extreme fat stimuli (all *p*s < 0.001). In a similar vein, the shape of extreme slim stimuli was judged more beautiful than that of normal slim stimuli independently of implied motion (all *p*s < 0.001). In contrast, the posture of extreme slim was judged more beautiful than that of normal slim stimuli for static (*p* < 0.001), but not for dynamic postures (*p* = 0.085). Thus, implied motion had a greater effect than body weight for the beauty judgments of the posture, but not of the shape, of normal and extreme slim bodies. Moreover, participants gave higher beauty VAS ratings in the posture than in the shape blocks for extreme and normal fat stimuli, independently of implied motion (all *p*s < 0.001). Conversely, the VAS scores were higher in the body shape than in the body posture block for static postures of normal and extreme slim models (all *p*s < 0.001) and for the dynamic postures of extreme (*p* < 0.001) but not normal slim models (*p* = 0.495).

### Influence of body size and motion on liking judgments of body shape and posture

**Figure 4:**
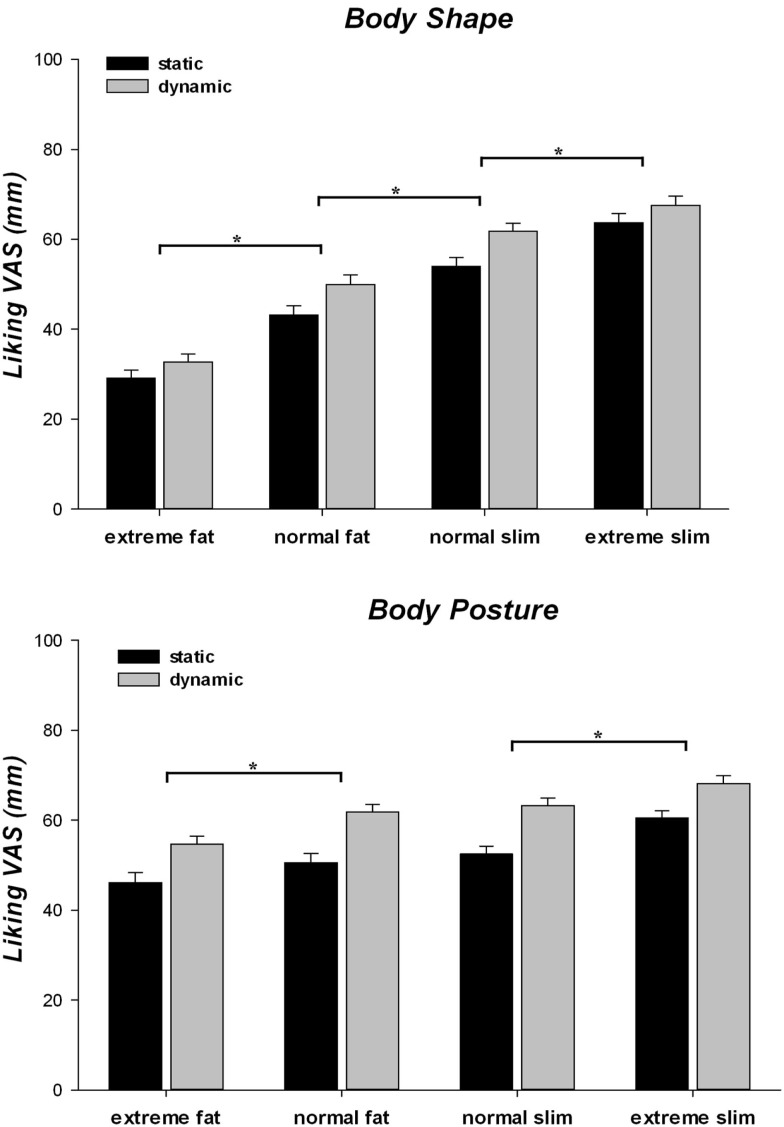
**Mean and standard error of mean for liking subjective rating of human models as a function of body shape and posture blocks, implied motion (static/dynamic) along the four categories of body weight (extreme fat/normal fat/normal slim/extreme slim)**. *p < 0.05.

The ANOVA on the judgments of how much the observers liked the models (Figure 4) revealed that all main effects were significant [all *F*s > 39.76; *p*s < 0.001; η ${\text{η}}_{p}^{2}$ > 0.335]. Indeed, overall participants liked more slim and dynamic stimuli than fat and static stimuli, respectively. Furthermore, the models’ postures were liked more than the models’ body shape. The two-way interaction between block and body weight was significant [*F*(3, 237) = 48.19; *p* < 0.001; η ${\text{η}}_{p}^{2}$ = 0.379]. *Post hoc* pair-wise comparisons demonstrated that in both body shape and body posture blocks participants liked more the normal than the extreme fat stimuli and the extreme than the normal slim stimuli (all *p*s < 0.001). However, they liked more the body shape (*p* < 0.001), but not the body posture (*p* = 0.231), of normal slim as compared to normal fat stimuli. Thus, the extreme slim stimuli received the highest and the extreme fat stimuli the lowest liking VAS scores in both blocks, but the effect of body weight was lower for the judgments of body postures than for the judgments of body shape. Indeed, while the liking judgments of both normal and extreme fat stimuli were lower in the body shape than in the body posture block (all *p*s < 0.001), no difference between the VAS ratings provided in the two blocks was observed for normal (*p* = 0.998) and extreme slim stimuli (*p* = 0.361). In addition, we found a significant two-way interactions between block and implied motion [*F*(1, 79) = 5.997, *p* = 0.017, η ${\text{η}}_{p}^{2}$ = 0.071]. *Post hoc* tests showed that dynamic stimuli were liked more than static stimuli in both blocks (all *p*s < 0.001) and that liking judgments were higher in the body posture than in the body shape blocks, independently from implied motion (all *p*s < 0.001). However, the increase of liking judgments for dynamic than static stimuli was stronger for the body postures (52.4 ± 1.56 vs. 61.98 ± 1.4) than for the body shape block (47.46 ± 1.29 vs. 52.97 ± 1.28). Finally, a significant two-way interaction between body weight and implied motion [*F*(3, 237) = 4.13, *p* = 0.007, η ${\text{η}}_{p}^{2}$ = 0.050] showed that dynamic stimuli were always liked more than static stimuli at all body weights (all *ps* < 0.001). For the extremely fat to normally slim weight, however, static bodies were liked more than their fatter dynamic versions (all *p*s < 0.001), whereas liking of dynamic normally slim stimuli was comparable with liking of static extremely slim stimuli (*p* = 0.621). This suggests that implied motion had a greater effect than body weight on the shape and posture liking judgments of models with a normally or extremely slim body.

### Predictors of perceived esthetic judgments for body shape and posture

Table 2 shows the summary statistics of standard multiple regression analyses conducted separately for attractiveness, beauty, and liking judgments of body shape and posture. Attractiveness judgment of body shape was associated with a positive perceived emotion, a well-proportioned body, a slim body, and perceived body similarity [whole model: adjusted *R*^2^ = 0.961; *F*(6, 137) = 589.27; *p* < 0.001].

**Table 2:** **Results of multiple regression analysis on affective and perceptual dimensions predicting attractiveness, beauty, and liking judgments for human models in body shape and body posture blocks**.

**RATING FOR BODY SHAPE**			
	**Attractiveness VAS**		
	***B***	***t***	***p*-level**
Athletic body	0.081	1.600	0.112
**Perceived emotion**	**0.327**	**8.423**	**0.000**
**Fatness**	**−0.170**	**−6.817**	**0.000**
**Proportioned body**	**0.205**	**3.397**	**0.001**
Healthy body	0.104	1.806	0.073
**Perceived body similarity**	**0.309**	**13.125**	**0.000**
	**Beauty VAS**		
	***B***	***t***	***p*-level**

**Athletic body**	**0.120**	**2.103**	**0.037**
**Perceived emotion**	**0.294**	**6.730**	**0.000**
**Fatness**	**−0.158**	**−5.644**	**0.000**
**Proportioned body**	**0.253**	**3.722**	**0.000**
Healthy body	0.127	1.960	0.052
**Perceived body similarity**	**0.213**	**8.032**	**0.000**
	**Liking VAS**		

	***B***	***t***	***p*-level**
Athletic body	**−**0.032	**−**0.449	0.654
**Perceived emotion**	**0.359**	**6.489**	**<0.000**
**Fatness**	**−0.159**	**−4.491**	**<0.000**
**Proportioned body**	**0.295**	**3.432**	**0.001**
**Healthy body**	**0.163**	**1.987**	**0.049**
**Perceived body similarity**	**0.198**	**5.899**	**0.000**
**RATING FOR BODY POSTURE**			
	**Beauty VAS**		

	***B***	***t***	***p*-level**
**Harmony**	**0.317**	**5.394**	**<0.001**
**Perceived emotion**	**0.628**	**9.903**	**<0.001**
Implied motion	0.062	1.244	0.215
Reproducibility of the movements	0.030	0.638	0.525
	**Liking VAS**		

	***B***	***t***	***p*-level**
**Harmony**	**0.446**	**7.811**	**<0.001**
**Perceived emotion**	**0.449**	**7.282**	**<0.001**
**Implied motion**	**0.150**	**3.089**	**0.002**
Reproducibility of the movements	0.081	1.783	0.077

*Significant p-values are marked as bold, for p < 0.05*.

The beauty evaluation relative to the body shape was associated with a high athletic VAS score, a positive perceived emotion, and a well-proportioned body [whole model: adjusted *R*^2^ = 0.951; *F*(6, 137) = 460.79; *p* < 0.001]. In addition, the beauty of body shape was related to low body fatness and perceived similarity, and high perceived body health was marginally significant (*p* = 0.052).

The multiple regression analysis of liking evaluations of models’ body shape revealed that perceived emotion was the most important predictor, followed by the effect of having a well-proportioned and a slim body [whole model: adjusted *R*^2^ = 0.921; *F*(6, 137) = 278.94; *p* < 0.001]. Furthermore, the liking for body shape was related to perceived body similarity and perceived body health.

The multiple regression analyses for body posture revealed that beauty judgments of models [whole model: adjusted *R*^2^ = 0.917; *F*(4, 139) = 396.7; *p* < 0.001] were associated with high harmony and positive perceived emotion judgments. In a similar vein, the analysis of liking judgments of postures revealed that harmony and positive perceived emotion were significant predictors [adjusted *R*^2^ = 0.922; *F*(4, 139) = 421.46; *p* < 0.001] as well as a perceived implied motion.

## Discussion

The present study investigated how the manipulation of body size and body motion cues influences the esthetic judgments of human body stimuli in a female sample. To this end, we asked participants to provide esthetic judgments on a series of virtual models depicted along four body weight categories, ranging from extremely fat and normally fat to normally slim and extremely slim figures. Furthermore, the different models were presented in static or dynamic postures, thus varying the implied motion evoked by each image.

First, we ascertained the success of our critical body weight manipulation and the potential influence of implied motion on body fatness perception. On the other hand, we searched for any influence of body fatness on the perception of implied motion evoked by images of static and dynamic postures. Then, we directly analyzed the influence of body weight and implied motion on attractiveness, beauty, and liking judgments of the models’ body shape and body postures. Finally, the perceptual and affective evaluative dimensions contributing to esthetic body perception of our set of experimental stimuli were further explored with multiple regression analyses. Four main findings resulted from the study.

First, we found that weight ratings of models parametrically reflected the manipulated body weight in the four categories used. Indeed, the extremely slim models were judged as the thinnest and the extremely fat stimuli were perceived as the heaviest figures. Second, body weight cue did not influence the amount of implied motion evoked by the postures, which always reflected the manipulated dimension of static vs. dynamic postures. Conversely, implied motion strongly affected the perceived body fatness of body stimuli, with the exception of extremely slim models, whose perceived heaviness was comparable for static and dynamic postures. Third, the esthetic perception of the models’ body, for all esthetic dimensions considered, was strongly affected by body size, with higher appreciation of thinner bodies, thus suggesting a clear-cut differentiation between esthetic evaluation of slim and fat bodies. Fourth, not only did implied motion strongly affect body esthetic perception, with higher appreciation of dynamic than static stimuli, but it also modulated the effects of body weight on esthetic judgments.

### Healthy women are reliable in rating body weight

When we explicitly asked participants to rate the heaviness of the body of other female and male individuals, their judgments increased linearly with the manipulated body weight for the four categories of extremely slim, normally slim, normally fat, and extremely fat models. These findings may suggest that healthy women are able reliably to rate others’ body fatness without being influenced by thin-ideal distortions. This may imply that, although the tendency of women to idealize thin body shapes is probably grounded in esthetic beauty experience, it is not associated with distortions in the perception of others’ body size. Furthermore, the current results are in keeping with those obtained by Brown and Slaughter (2011), who showed that participants were reliable in their objective perceptions of female body normality and, at the same time, they consistently rated extremely slim bodies as more attractive than normal bodies. These data are in keeping with the notion that the discrepancies between body ideals and perceived body fatness rather than disorders of body perception are probably associated with body image disturbances (Thompson and Stice, 2001; Bessenoff, 2006).

Although our participants were sampled from a non-clinical population, several studies have shown that body dissatisfaction is pervasive among young women (Kenardy et al., 2001; McCabe and Ricciardelli, 2001; Abbate-Daga et al., 2007). A discrepancy between reliable others’ body perception and self- vs. metacognitive representation of one’s own body is in keeping with studies by Jansen et al. (2005, 2006). These authors investigated the congruency between the evaluations of the attractiveness of the participants’ body provided by the participants or by other individuals. They found that self-referred evaluations of healthy individuals were more positive than those provided by others, whereas those of patients with anorexia nervosa were more coherent with the judgments provided by others (more objective). These results suggest the existence in healthy individuals of a self-serving bias that affects the perception of one’s own but not others’ body.

### Implied motion affects body size perception

We found an influence of implied motion of human bodies on heaviness judgments that was lower for the dynamic than for the static postures of extremely fat, normally fat, and normally slim body models. Only judgments of extremely slim bodies were insensitive to implied motion, as no difference was found between extremely slim models shown in dynamic and static postures. This may be related to the notion that exercise is regarded as important health behavior for most people. The popular media often promote exercise as a means of achieving the thin and firm current body ideal (Homan, 2010) and, indeed, the proportion of exercise-related references in women’s magazines has steadily increased with the frequency of exercise articles, which have now surpassed the frequency of diet articles (Wiseman et al., 1992).

Despite the strong influences of implied motion on body weight perception, the perception of the postures’ implied motion did not change according to body weight. This suggests that whereas the body motion perception system can extract motion information from the relative positions of body parts regardless of their shapes, the cognitive system involved in the perception of body shape is influenced by motion-related body configurations. Indeed, the morphology of body parts changes dramatically during movement and the body shape perceptual system must continuously take into consideration whether the body is relaxed or in motion. Furthermore, motor-related tasks require the representation of the sensory consequences of the movements and motor structures may feed back into the visual cortex and trigger the activation of a visual representation of the moving body part. Neuroimaging studies have, indeed, demonstrated that neural activity of EBA is modulated by self-initiated movements (Astafiev et al., 2004; Kühn et al., 2011) and by the observation of moving vs. static displays of human bodies (Kable and Chatterjee, 2006; Piefke et al., 2009). Thus, implied motion perception may affect the activity of occipito-temporal areas devoted to body shape processing and influence the perceived heaviness of moving bodies.

### “Thinner is better”

In keeping with the thinness ideal of beauty, we expected that young women would attribute higher attractiveness, beauty, and liking judgments to slim rather than to fat models. This was overall supported by our data, confirming that a thin beauty ideal was shared by our sample. Indeed, extremely fat body shapes were judged as the most repulsive, the ugliest, and the most disliked stimuli and the extremely slim body shapes as the most attractive, beautiful, and liked. These findings support the notion of social pressure, probably mediated largely by media exposure, leading to an internalization of the thin-ideal, which is partially independent of sexual arousal or attractiveness (Collins, 1991; Feingold and Mazzella, 1998; Kostanski and Gullone, 1998; Pine, 2001; Truby and Paxton, 2002).

Previous studies have shown that the idealization of thinness in relation to one’s own body is more pronounced in females than in males (Fallon and Rozin, 1985; Feingold and Mazzella, 1998), whereas dynamicity and healthy are emphasized more for males (Brown et al., 2005). On the other hand, other studies have reported no gender differences when adults rate female body attractiveness (e.g., Tovée and Cornelissen, 2001; Winkler and Rhodes, 2005; Smith et al., 2007a,b), suggesting that, in keeping with our data, both men and women value thinness and dynamicity in adult male and female bodies (Cornelissen et al., 2009; Brown and Slaughter, 2011).

### “Moving is better”

Esthetic evaluations of human bodies were strongly affected by implied motion. This occurred not only when participants evaluated body postures, but also when they evaluated body shapes. In other words, models belonging to the same body weight category were judged more attractive, beautiful, and likeable when presented in a dynamic than in a static posture. As dynamic bodies were perceived as thinner than static stimuli, the effect of implied motion on body shape esthetic judgments was probably mediated by the changes in perceived body size. On the other hand, that more dynamic postures received better esthetic judgments is in keeping with studies of esthetic appreciation of dance moves (Calvo-Merino et al., 2008; Cross and Ticini, 2012), showing that more dynamic stimuli involving whole body movement with significant displacement of the body in space received better liking judgments than more static stimuli.

Finally, the contribution of perceptual and affective evaluations to the esthetic appreciation of others’ bodies was confirmed by regression analysis for esthetic attractiveness, beauty, and liking judgments of body shape. Indeed, esthetic ratings of human body shape were predicted by perception of an athletic and proportionate body as well as by perceived positive emotion. Similarity may be judged as attractive or beautiful because it has many adaptive functions, including facilitating dyadic interactions, fostering a sense of familiarity and safety, and validating individuals’ self-concepts (Byrne, 1971). All in all, our findings suggest that women’s esthetic evaluation of bodies is determined by the additive contributions of perceptual and affective components of body representations. Altogether, these representations may foster esthetic experience and express the subjective notion of beauty. Thus, the esthetic appreciation of the body is not only driven by the physical categorization of the body as slim and well-proportioned, but also by the positive emotions evoked by its movements.

### Limitations and future directions

There are some limitations to this research which warrant consideration. First, it is possible that the esthetic appreciation of human body shapes and postures may be different in male observers. As only women were included in this research, we cannot exclude the possibility that men’s judgments of beauty, liking, and attractiveness are influenced by weight variation and implied motion differently from women’s. Furthermore, as our study design does not allow us to test how the same participant responded to male and female models, we cannot determine the influence of perceiving opposite vs. same sex individuals. Research into the esthetic perception of human body in both genders is generally lacking and an understanding of how objective and subjective esthetic measures of males’ and females’ body shape and size are related to body satisfaction and disordered eating would be an important area for future study.

Second, all participants were university students (primary teaching and professional education) who completed the study in the university setting. Thus, the use of a university sample of restricted age range may limit the generalizability of our results to the wider population. Future studies should investigate whether the results can be generalized to non-student populations, non-Western women, older women, and men. In particular, it would be useful for future research to investigate body esthetic perceptions in children and adolescents of bodies from their own age groups, in order to document more thoroughly the pervasiveness of the thin-ideal in esthetic experience.

Another limitation is that we did not take direct measurements of the participants’ BMI. Indeed, a self-reported measure of BMI could be affected by possible body image distortions, which may be present in otherwise healthy individuals. Furthermore, given that judgments of one’s own body can be influenced by fluctuating affective, physiological, and cognitive states (Tiggemann, 1996; Farrell et al., 2005), it may be useful in future studies to measure these states in order to determine whether they also affect esthetic judgments of others’ bodies. Although, our experimental stimuli constitute a significant improvement on the hand-drawn silhouettes typically used in this research, they still lack the direct realism of life-size images.

The specific behavioral association between esthetic judgments of body shape and posture with implied motion and weight variation obtained in this study could stimulate future data-driven explorations of the neural underpinnings of the esthetic experience. Monitoring eye movements in future research could provide further information on the specific body cues used during esthetic perception of the body. Neurophysiological and neuroimaging techniques can be used to explore whether specific brain areas of the dual-route model, such as EBA and premotor cortex, respectively involved in the local or configural processing of the body, play specific and complementary roles in the esthetic perception of body form and body actions (Calvo-Merino et al., 2010).

All in all, our experiment indicates that esthetic body perception is affected both by socio-cultural attitude to idealized thinness and by the perceptual features of visual variants such as implied motion. Future research investigating the reciprocal influence of cultural and perceptual dimensions of body image on body esthetic perception may help to provide psychologists and educators with valuable information for implementing educational programs aimed at improving body image satisfaction among adolescent populations.

## Conflict of Interest Statement

The authors declare that the research was conducted in the absence of any commercial or financial relationships that could be construed as a potential conflict of interest.
